# The Efficacy of Telehealth Versus In-Person Management Delivery in Adult Patients with Obesity

**DOI:** 10.3390/healthcare12212190

**Published:** 2024-11-04

**Authors:** Rawan A. Alolayan, Dara A. Aldisi, Danish S. Hussain, Nora Alafif, Mahmoud M. A. Abulmeaty

**Affiliations:** 1 Department of Community Health Sciences, College of Applied Medical Sciences, King Saud University, Riyadh 11362, Saudi Arabia; 443204041@student.ksu.edu.sa (R.A.A.); daldisi@ksu.edu.sa (D.A.A.); nalafeef@ksu.edu.sa (N.A.); 2 Biochemistry Department, College of Science, King Saud University, Riyadh 11451, Saudi Arabia; shussain@ksu.edu.sa

**Keywords:** obesity, obesity management program, telehealth

## Abstract

Background: The effectiveness of telehealth in managing obesity in Saudi patients is still under investigation. This study compared the effectiveness of telehealth and in-person obesity management programs for adults. Methods: This clinical trial involved 62 adults, 29 receiving in-person intervention at the clinic and 33 receiving telehealth via videoconference calls. Anthropometric measurements, biochemical parameters, and dietary and lifestyle habits were assessed at baseline and after 12 weeks. Patients have been educated about goal-setting, healthy eating behaviors, personalized meal plans, and increasing physical activity levels. Results: It showed that 45% and 49% of the in-person and telehealth groups lost more than 5% of their initial body weight. Weight, % body fat, and waist circumference were significantly reduced in the in-person and telehealth groups (*p* < 0.001). No significant differences between the groups were found in the parameters mentioned above. Within-group analysis showed that light physical activity levels improved in both groups significantly (*p* < 0.001), and the moderate physical activity level improved significantly among the telehealth group (*p* < 0.039). No significant differences were observed between the groups regarding physical activity level, blood pressure measurement, and biochemical markers, except for the RBC blood level (*p* = 0.026). The telehealth group had significantly higher attendance rates for counseling sessions (97% vs. 75% for the in-person group, *p* < 0.01). Participants’ dropout rates were higher for the in-person group 21%, compared to the telehealth group 13%. Telehealth participants’ satisfaction regarding the benefits of the obesity management program in losing weight was higher at 87% compared to the in-person group at 76%. Conclusions: In conclusion, applying a telehealth obesity management program can support patients struggling with obesity who may have limited access to traditional healthcare services, while ensuring that telehealth care replicates the quality of in-person care.

## 1. Introduction

Obesity, a persistent medical condition, poses a significant healthcare challenge in the Kingdom of Saudi Arabia, impacting over 20% of the population [1]. No single disease has negative health implications, affecting the whole-body systems and organs and subsequently increasing morbidity and mortality, such as obesity [2,3]. Even a modest reduction in weight of around 5% is a clinical benchmark recognized to produce clinically meaningful health improvements in obesity-related comorbidities [4]. As per the Saudi clinical practice guideline for the management of obesity, the cornerstone for obesity therapy is lifestyle modification, which is usually achieved through multi-model lifestyle intervention that includes behavior therapy, medical nutrition therapy, and physical activity [4,5,6].

The direct healthcare costs for patients with obesity are 30% higher compared to non-obese counterparts. In Saudi Arabia, if no serious actions are taken, the cost of overweight and obesity is expected to increase drastically from USD 19 billion (2.42% GDP) in 2019 to USD 78 billion (4.16% GDP) by the year 2060 [7]. The healthcare sector in Saudi Arabia faces the challenge of delivering high-quality healthcare to the entire population and improving access to healthcare services across the country’s vast size and diverse terrain. Implementing telehealth obesity management intervention may bridge the gap [8,9,10,11,12,13,14,15]. Digital health is considered a key driver in the recent healthcare system reformation: improving the quality of care, customer experience, access to specialized medical professionals, and promoting preventive practices. Therefore, the healthcare cost of obesity should be reduced to support the government’s economic reformation through Vision 2030 of Saudi Arabia [16,17,18,19,20].

Although telehealth interventions to treat obesity are in their infancy, studies show that telehealth is an essential and effective tool for obesity therapy [21,22]. The recent Coronavirus disease 2019 (COVID-19) pandemic speeds up the adoption of telehealth to provide effective and urgent medical care for patients [23]. A powerful tool in telehealth interventions is using digital self-monitoring devices (e.g., activity tracker, dietary intake, and body composition Wi-Fi scale). It lowers the burden of self-monitoring and increases patient engagement and empowerment [16]. It enhances compliance and enables more regular clinical engagements, thereby increasing the likelihood of achieving success [16]. Virtual consultations with healthcare providers help to discuss progress and challenges and receive personalized advice on healthy lifestyle modification. By applying telehealth, healthcare providers in Saudi Arabia can more effectively reach and support patients struggling with obesity, who may have limited access to traditional healthcare services. However, the acceleration of the adoption of telehealth technology has raised a great concern regarding the efficacy of telehealth care, i.e., whether it replicates the quality of in-person care or not [24,25].

This study evaluated the short-term effectiveness of telehealth obesity management intervention compared to in-person (usual care) programs regarding changes in weight, % body fat, waist circumference, dietary and lifestyle habits, physical activity level, blood pressure, biochemical markers, patients’ adherence, and satisfaction. To the best of our knowledge, our study will be the first to tackle telehealth interventions among adults with obesity in Saudi Arabia and to compare obesity management interventions by telehealth and in-person methods.

## 2. Materials and Methods

### 2.1. Study Design and Settings

This was a non-randomized controlled trial. Participants for the study were recruited through social media announcements (email, Twitter, WhatsApp^®^ groups) and billboard announcements in waiting areas at governmental hospitals and academic institutes (King Khaled University Hospital, King Saud University, King Saud bin Abdulaziz University). The study staff assessed the eligibility of participants. Participants were divided according to the type of intervention: group I, which was in person, and group II, which was telehealth. The selection of patients was according to patient convenience. At the baseline visit, both groups were required to visit the Clinical Nutrition clinic at the College of Applied Medical Sciences (CAMS) at King Saud University in Riyadh, where a detailed explanation of the study was provided to all subjects, and consent forms were signed. The privacy and confidentiality of patients were completely protected. All participants were provided with a wireless body weighing and body composition scale (Anker smart scale P2, Changsha, Hunan, China) along with its corresponding Eufy Life application, as well as a wearable activity tracker for physical activity monitoring (Sound PEATS Smart Watch, Chicago, IL, USA) along with its Sound PEATS SPORTS application. These applications facilitated virtual communication. At the baseline visit, the researcher installed, activated, and demonstrated the scales’ usage and applications to the participants and followed up to troubleshoot any technical issues. The two applications support the Arabic language, and before buying the tools, the research tested their convenience for the patients. Additionally, the researcher ensured that the patients had the Zoom application for video conferencing and the WhatsApp application for providing educational materials, troubleshooting any technical issues, and two-way text messaging between visits. The King Abdulaziz International Medical Research Center (KAIMRC) review board approved the study protocol with the reference IRB No. IRB/0322/23.

### 2.2. Sample Size Calculation

Alencar et al. [10] reported the efficacy of a telemedicine-based weight loss program with video conference health coaching support to achieve weight reduction with an estimated effect size of 1.173. Using a more conservative effect size of 0.8 and achieving the study’s power of 80%, the required sample size is at least 26 per arm at 95% CI. This study recruited 62 participants.

### 2.3. Subjects

The study involved a total of 62 adults aged 18–65 years. Participants were selected based on specific inclusion and exclusion criteria, which included a BMI of 30 or more and having a smartphone. The exclusion criteria for the study encompassed several categories. These included individuals with cognitive difficulties and women who were within 12 months postpartum. Additionally, patients with various medical conditions such as psychiatric disorders, diabetes, hypothyroidism, gout, phenylketonuria, celiac disease, chronic kidney disease, cancer, eating disorders, chronic obstructive pulmonary disease, human immunodeficiency virus/acquired immunodeficiency syndrome, heart failure, stroke, heart attack, and those under comfort care were excluded. Furthermore, individuals with a history of metabolic/bariatric surgery and those who had participated in a weight loss program and lost more than 4.5 kg in the previous six months, individuals with specific dietary preferences (e.g., vegetarian and keto diets), and patients on weight loss medications or on certain medications known to impact weight were also excluded from the study.

### 2.4. Data Collection

A self-reported questionnaire was used to collect participant information, including socio-demographic data, past medical history, and medication intake. The trained research staff conducted the anthropometric assessment and collected fasting blood samples at the beginning of the study and after the intervention. During the first visit (60 min. per visit), the participants underwent an examination of blood pressure (in millimeters of mercury), weight (in kilograms), height (in centimeters), waist circumferences (in centimeters), and % of body fat using standard procedures. The body mass index (BMI) was calculated by dividing the weight in kilograms by the square of the height in meters. Furthermore, the interviewer-administered interview questionnaire includes socio-demographic data, past medical history, and medication intake. Additionally, a lifestyle and food frequency questionnaire was used, which included four sections to gather information on dietary habits, sun exposure, exercise routines, and sleep patterns [26]. Moreover, participants were asked to document any food or drink intake for three days and were educated on how to estimate the serving size. The nutrient analysis of this three-day food record was carried out with the Food Processor^®^ software version 11.1 (Esha Research Inc., Salem, OR, USA), which is used to analyze the dietary components. Most of the Arabic local dishes were available, and some local dishes were missed and their recipe ingredients were added.

Additionally, participants were asked to document their daily steps for three months on a special sheet. The fasting blood samples collected at the beginning of the study and after the intervention were promptly transported to the laboratory, where they were stored under appropriate conditions for biochemical analysis. The analysis assessed CBC, glucose, vitamin D, lipids, renal, and hepatic profiles using standard routine kits and an automated biochemical analyzer (Fujifilm, Tokyo, Japan). All biochemical analyses were performed at the College of Applied Medical Sciences, King Saud University. Moreover, we assessed participants’ readiness to change. After 12 weeks of the intervention (visit No. 7, 60 min.), both groups came to the dietitian clinic, and the same above-mentioned parameters were measured again. Additionally, patients’ satisfaction was assessed, and the participants returned the wireless body weighing scale and the wearable physical activity tracker.

Both arms received similar content of 5 follow-up visits (30 min. per visit), with intervals of 2 weeks between the visits [4]. The in-person group came individually to the dietitian clinic, and the telehealth group via videoconference call (individually). Every two weeks, all patients received a reminder text message the day before their appointment via WhatsApp^®^ application to send a copy of the two weeks’ daily step count record and measure their weight and % body fat, then send it to the researcher. Detailed information on the procedures for each visit is listed in Table S1.

### 2.5. Obesity Management Protocol

All participants were given (A) a wireless body weighing and body composition scale and (B) a wearable tracker for physical activity monitoring. Both arms received a similar interventional program; there was no variation in the intervention between the in-person and telehealth groups. All participants received similar nutrition education flyers, but the way of delivering the information varied from one patient to another according to the patient’s needs. Except for the 2nd visit, a personalized meal plan based on the Mifflin St Joer equation [27], with a targeted energy deficit of 600 Calories per day, which, in theory, should produce a 0.5 kg weight loss per week, was provided for each patient [4]. The program included seven visits over three months. Short-timeframe studies of 12 weeks can still produce clinically meaningful health improvements in obesity-related comorbidities [4,28,29,30,31]. During the five follow-up visits, the weight loss prescription included personalized nutritional assessment, goal-setting, weight loss progress, daily step count discussions, educational programs consisting of eating behaviors and mindful eating (stimulus control, identification of high-risk situations for relapse, hunger, speed of eating, emotional/pleasure eating, late-night eating, and portion control) [32], the eat well plate (Mediterranean diet), personalized meal plan, how to achieve satiety and encouragement to drink 2 L of water per day, healthy snacks and healthy food choices at restaurants, what to do after eating an unhealthy meal, and weight maintenance tips. Moreover, patients were encouraged to do aerobic physical activity for 150 to 300 min per week [33], increase their daily living activities and the number of steps per day to 5000–10,000 steps per day [34], and sleep 8 hours per day [35]. The same dietitian conducted all sessions, and similar scripts were used to ensure consistency between the two groups. All participants had 24-h, 7-day mobile hotline support from the research team.

### 2.6. Statistical Analysis

Statistical Package for Social Sciences (SPSS version 21.0) was used to analyze the results. The Shapiro–Wilk test was used to test the normality of the data. Descriptive statistics, including Mean ± Standard Deviation (SD), were used to present normal variables, whereas Median (Quartile1–Quartile 3) was used to present non-normal variables. Frequencies and percentages were used to present categorical variables. To identify the significance of the differences between in-person and telehealth groups at the baseline level, independent sample t-tests or Mann–Whitney U tests were employed for normally distributed and non-normally distributed variables, respectively. Chi-square or Fisher’s exact tests were used for categorical variables to identify significant frequency differences between in-person and telehealth groups. Non-normal variables were log-transformed before using parametric testing to achieve approximate normality. Repeated measures ANOVA (which gives multivariate analyses for the repeated measured variables) and generalized estimating equations (GEE) were used to identify differences within and between in-person and telehealth groups for continuous and categorical variables, respectively. A significance level of 0.05 is considered significant.

## 3. Results

### 3.1. Baseline Characteristics of the Participants

A total of 62 adult participants enrolled in this study, of which 29 were assigned to receive an in-person obesity management program, and 33 were assigned to receive a telehealth obesity management program. Gender distribution, monthly income, educational status, and marital status profiles were similar in the two groups. Most participants were non-smokers, and their distribution was similar across the two groups. Furthermore, no significant differences were found according to the morbidity status of the participants between the two groups (Table 1). Table 1 also shows the participant’s readiness for change in the telehealth and in-person groups. No differences were observed regarding participants’ readiness for change, where most participants were at the preparation stage while the remaining were at the action stage.

The mean age of the participants was 31.7 ± 11.8 and 32.3 ± 11.6 in the in-person and telehealth groups, respectively. No significant differences in weight, BMI, waist circumference, and % body fat were observed among the two groups at baseline. Furthermore, both groups also showed similar fasting glucose and lipid profiles. In contrast, significant differences were found in MCV, MCH, RDW, and potassium levels between the groups (Table 2).

### 3.2. Changes in Anthropometrics, Glucose, Lipid, Renal, and Hepatic Profiles

Table 3 shows the baseline and after 12 weeks of the outcome of the obesity management program in the telehealth and in-person groups. No significant differences were observed in anthropometrics, glucose, lipid, renal, and hepatic profiles between groups. Furthermore, no significant interactions were observed. However, post hoc comparisons showed that participants who received an obesity management program in person experienced a significant reduction in weight from 95.8 ± 13.9 to 90.7 ± 14.0 (*p* < 0.001), BMI from 35.1 ± 4.2 to 33.2 ± 4.1 (*p* = 0.001), waist circumference from 108.8 ± 11.3 to 102.4 ± 11.3 (*p* < 0.001), and % body fat from 42.6 ± 5.7 to 41.0 ± 6.0 (*p* < 0.001). In comparison, participants who received telehealth intervention also experienced significant reduction in weight from 95.7 ± 21.4 to 91.2 ± 21.2 (*p* < 0.001), BMI from 35.6 ± 5.4 to 33.9 ± 5.4 (*p* < 0.001), waist circumference from 107.0 ± 15.6 to 101.8 ± 15.0 (*p* < 0.001), and % body fat from 43.0 ± 4.8 to 41.7 ± 4.9 (*p* < 0.001). Moreover, 45% of those who were enrolled to receive an obesity management program in person experienced at least 5% weight loss compared to 49% who received a telehealth obesity management program, and no significant difference was observed between the groups. Furthermore, no significant changes were observed in the participants’ fasting blood sugar or lipid profile within groups. While a significant increase in BUN level was observed at the end of the study compared to the baseline in the telehealth group only, no other significant changes were observed in the participants’ hepatic profile, uric acid, or vitamin D within groups. Moreover, there were no significant changes in blood pressure measurements from the baseline and after 12 weeks of the obesity management intervention within the groups.

### 3.3. Changes in Lifestyle Parameters

Table 4 shows the participants’ physical activity levels at baseline and after 12 weeks within and between the in-person and telehealth groups. No significant differences were observed in physical activity levels between groups. Furthermore, no significant interactions were observed. Within-group analysis showed that participants who received an obesity management program in person experienced a significant increase in light physical activity levels. At baseline, 35% reported being physically active in light exercises for at least 30 min more than once a week; however, at the end of the study, 86% reported being physically active in light exercises for at least 30 min more than once a week, which was significant (*p* < 0.001). Although the number of participants doing moderate and vigorous physical activity increased at the end of the study compared to the baseline, changes were not statistically significant. Within-group analysis showed that participants who received telehealth obesity management programs experienced significant increases in light and moderate physical activity levels. Concerning light physical activity at baseline, 39% reported being physically active for at least 30 min more than once a week; however, at the end of the study, 79% reported being physically active for at least 30 min more than once a week which was significant (*p* < 0.001). Similarly, group analysis showed that, at baseline, 9% reported being physically active in moderate exercises for at least 30 min more than once a week; however, at the end of the study, 30% reported being physically active in moderate exercises for at least 30 min more than once a week which was significant (*p* = 0.039).

Table 5 shows changes in the sleeping patterns of the participants at the end of the study between the telehealth and in-person obesity management groups. No significant differences were observed in sleeping habits between groups. Furthermore, no significant interactions were observed. Within-group analysis showed a significant decrease in total sleeping hours among the in-person and telehealth groups (*p* = 0.002 and *p* = 0.01, respectively). Moreover, the within-group analysis showed that the duration of sleeping hours at night significantly decreased among the in-person and telehealth groups (*p* = 0.002 and *p* = 0.01, respectively). Additionally, we observed a significant decrease in the average time (in minutes) spent from going to bed to falling asleep among the in-person and telehealth groups (*p* = 0.04 and *p* = 0.01, respectively). Furthermore, Table 5 also shows the macronutrient intake of the participants using a food frequency questionnaire at baseline and after 12 weeks within and between the in-person and telehealth groups. No significant differences were observed in eating habits between groups. Furthermore, no significant interactions were observed. The data showed that there is a significant increase in the mean intake of fruits in both in-person (*p* = 0.012) and telehealth groups (*p* = 0.005). Although mean vegetable intake also increased, this increase was not significant in the in-person group (*p* = 0.083) as compared to the telehealth group, where it increased significantly (*p* < 0.001). Furthermore, mean carbohydrate intake also decreased significantly in the in-person (*p* = 0.022) and telehealth group (*p* = 0.036). However, no significant difference was found in fat intake within and between the groups.

The macro- and micronutrient compositions of the dietary intake based on three days’ food record at baseline and 3 days’ food record after 12 weeks within and between the two groups are presented in Supplementary Tables S2–S4. No significant differences were observed in physical activity levels between groups, with the exception of the rare intake of vitamin A, which was significantly higher in the telehealth group (*p* = 0.029). Furthermore, no significant interactions were observed except for caffeine intake, which reduced significantly in the telehealth group post-intervention (*p*-interaction = 0.015). Within-group analysis revealed that the participants who received an obesity management program in person experienced a significant reduction in energy from fat (*p* = 0.043), energy from saturated fat (*p* = 0.026), amount of fat (*p* = 0.043), and amount of saturated fat (*p* = 0.028) as shown by within-group analysis. In comparison, the telehealth group also experienced a significant reduction in energy intake (*p* = 0.049), energy from fat (*p* = 0.021), energy from saturated fat (*p* = 0.036), amount of sugar (*p* = 0.025), amount of added sugar (*p* = 0.012), amount of fat (*p* = 0.022), amount of saturated fat (*p* = 0.041), amount of caffeine (*p* = 0.001), and amount of fluoride (*p* = 0.025) (Tables S2–S4).

Supplementary Table S5 shows the eating patterns of the participants at baseline and after 12 weeks of the study in the in-person and telehealth groups. Participants in the telehealth group reported significant changes in their timing of carbohydrates and sugar consumption. Post intervention, the number of telehealth participants eating carbohydrates and sugars at dinner meals decreased significantly. In contrast, the number of participants eating the largest portion of carbohydrates and sugars increased in the morning breakfast (*p* = 0.027). Similarly, post intervention, the number of participants eating fats at dinner decreased significantly, whereas those eating the most significant portion of fats increased at lunch meals (*p* = 0.027). Sugar intake also declined substantially in the in-person group (*p* = 0.037) only. Furthermore, both groups showed a significant increase in mean cups of water consumption (*p* = 0.01), and participants receiving in-person intervention also reported a significant increase in water intake before the start of the meal (*p* = 0.006).

### 3.4. Self-Adherence and Satisfaction

Participants’ attendance rates for the counseling sessions were significantly higher for the telehealth group, 97% compared to 75% for the in-person group (*p* < 0.01). Furthermore, adherence to biweekly self-weighing was higher among the telehealth group, 100%, compared to 96% for the in-person group (*p* = 0.282). Similarly, adherence to wearing the step counter was higher among the telehealth group, 75%, compared to the in-person group, 65% (*p* = 0.375). Participants in the in-person group had a higher dropout rate of 21.6% compared to the telehealth group of 13% (*p* = 0.332).

Participants were asked to rate their satisfaction with the programs using three questions: (a) “was overall satisfied with the allocated obesity management program”, (b) “feel the assigned dietitian was helpful to them?” and (c) “feel allocated obesity management program was helpful to lose weight?”. Over 95% of participants in both groups reported being very satisfied or satisfied with the question “was overall satisfied with the allocated obesity management program” (*p* = 0.763). For the second question, “Feel the assigned dietitian was helpful to them?”, all of the participants in both groups reported being very satisfied or satisfied (*p* = 0.322). For the third question, “Feel allocated obesity management program was helpful to lose weight?”, more than 87% of the telehealth group participants were very satisfied, compared to 76% of the in-person group (*p* = 0.604).

## 4. Discussion

The results of this study highlight the effectiveness of in-person and telehealth obesity management programs in promoting weight loss and improving lifestyle behaviors among participants. Interestingly, while there were no significant differences in baseline characteristics between the two groups, both groups experienced significant reductions in weight, BMI, waist circumference, and body fat percentage after the 12-week intervention.

If telehealth interventions replicate the components of face-to-face interventions, the potential benefit lies in reduced contact time, rendering it potentially more cost effective [16,23]. Several studies have investigated the effectiveness of telehealth intervention compared to traditional, in-person approaches for treating obesity [8,9,10,11,12,13,14].

Our result agrees with Marra et al.’s randomized control study that included 59 men with obesity aged 40–70 years. The dietitian delivered three virtual medical nutrition therapy sessions and nine telephonic nutrition counseling sessions for the telehealth group only; all participants had three in-person visits that did not include education about the physical activity component. Results revealed insignificant differences in weight loss between the telehealth and in-person groups over the three-month trial period [8]. On the contrary, Barnason et al. conducted a randomized control study over six months among 43 rural cardiac subjects. The telehealth group had better outcomes related to receiving more intensive weight management intervention that included two telehealth coaching sessions by the research nurse and 36 recorded telehealth sessions. In contrast, the in-person group received brief management for overweight and obese participants, the usual care of a post-cardiac revascularization program [9]. Additionally, Alencar and his group conducted a single-blinded randomized control study on a small sample size of 25 participants over 12 weeks. They found significant differences in weight loss between the telehealth and the in-person groups. The telehealth group had 15 videoconference sessions compared to 4 sessions for self-monitoring activity with no health coaching for the control group [10]. Moreover, a larger sample size study included 351 adults with diabetes, hypertension, and dyslipidemia, conducted over a longer time period of 12 months. The researchers reported significantly better weight loss, favoring the telehealth group, whereas the in-person group (usual care) received self-help materials, newsletters, and a list of community resources for weight management. Conversely, the telehealth group received 18 coaching calls from the dietitian [11].

About half of the participants from each group reached clinically meaningful weight loss of 5% at the end of the study, which is considered a clinical benchmark recognized to produce clinically meaningful health improvements in obesity-related comorbidities [4]. However, a larger proportion of participants in the telehealth group achieved clinically meaningful weight loss than the in-person group, 49% vs. 45%, respectively, without statistically significant difference between the two groups. Similarly, the above-mentioned Barnason et al. study showed that 70% of the telehealth group and 41% of the in-person group participants achieved a clinically meaningful weight loss of 5% without significant difference between the groups after 12 weeks of the intervention [9]. The longer-duration Bennett et al. study showed that 43% of the telehealth group and 6% of the in-person group achieved clinically meaningful weight loss (more than 5%) after 6 months of the intervention. The telehealth group received 18 coaching calls, and the in-person group (usual care) received self-help weight management materials only. [11]. Alencar et al. study with a small sample size of 25 participants reported that 70% of the telehealth group achieved clinically significant weight loss, compared with only 8% of the in-person group. This huge difference can be justified by the variation in the number of counseling sessions, 15 for the telehealth group versus 4 sessions for the control group [10]. Few interventions have attempted to compare telehealth and in-person obesity management programs, and none have implemented similar interventions in obesity management programs like those tested in our trial. Our study indicates that telehealth approaches hold promise for extending the reach of more patients with obesity.

There were few between-group differences regarding dietary intake. There was a statistically significant reduction in energy intake among the telehealth group only, but not for the in-person group. This difference between groups can be interpreted by the higher attendance rate of the telehealth group. Our findings were inconsistent with those of a randomized control study that reported a significant reduction in energy intake among the telehealth and in-person groups. This difference found in the previously mentioned study compared to our study with respect to energy intake can be justified by the delivery of dissimilar obesity management interventions in both the telehealth groups and in-person group: 15 versus 3 sessions, respectively [8].

In our study, participants in both groups also consumed significantly lower total and saturated fats post-intervention. However, no significant differences were observed between groups. These results were consistent with another study [8], which also detected no significant differences between groups concerning self-reported dietary behaviors.

Additionally, our study gave more details about dietary intake that were unavailable in the previously mentioned studies; our findings show that telehealth interventions were more effective than in-person interventions in decreasing sugar and added sugar intake. The higher attendance rate among the telehealth group can explain this difference between groups. Moreover, Lugones and his group conducted a randomized control study on patients with obesity, where all patients received brief healthy lifestyle advice, and the mobile health (mHealth) intervention group received a smartphone application for self-recording the daily diet and activity tracker wristband without further counseling sessions with healthcare personal. The research revealed a significant reduction in energy, fat, saturated fat, and sugar intake among the two groups over 12 months [13]. Lugones study showed the effectiveness of self-monitoring tools. However, there were no personalized counseling sessions with a healthcare provider similar to our study. Self-monitoring tools alone are not usually enough to foster healthy behavioral change [15].

In our study, we assess the participants’ dietary habits. Eating carbohydrates and sugars at dinner decreased significantly, whereas eating carbohydrates and sugars increased at breakfast among the telehealth group only. Similarly, eating fats at dinner decreased significantly, whereas eating fats increased at lunch among the telehealth group only. The adjustments in nutrient intake observed in this study suggest that participants could make sustainable changes to their lifestyle habits.

Our study’s findings revealed that in-person and telehealth interventions observed increased physical activity levels compared to baseline levels. The participants in the in-person and telehealth groups experienced significant increases in light physical activity levels over the 12-week trial period by 52% and 39%, respectively. The increase in physical activity levels indicates that both interventions successfully motivated participants to exercise more. Moreover, a significant increase was observed for moderate physical activity levels among the telehealth group by only 21%, without significant differences between the two groups regarding light and moderate physical activity levels. Our findings were inconsistent with the study by Barnason et al., which reported insignificant changes in physical activity levels among the two groups; those findings can be justified by the lack of physical activity level measurement at the beginning of the study [9]. In addition, our findings were consistent with the Alencar et al. study that showed a significant increase in average weekly steps among the telehealth group only, but this study has limitations of a small sample size and did not provide a similar intervention for the control group as we mentioned earlier [10].

As expected, the participants’ attendance rates for the counseling sessions were significantly higher for the telehealth group, at 97%, in comparison to 75% for the in-person group. Our study was conducted in the capital city of Saudi Arabia, Riyadh with a population of 10.5 million, which experiences high traffic congestion, which may affect the in-person participant’s attendance rates to the clinic. Likewise, the findings of the above-mentioned studies showed a higher attendance rate among the telehealth group [10,11,14].

A fundamental part of the trial was the usefulness of the self-monitoring tools (wearing a daily activity tracker, the 3-day food record at baseline and after 12 weeks, and using a body composition Wi-Fi scale). Participants can monitor their progress towards their goals and discuss their progress during the biweekly nutritional sessions where the dietitian reinforces them for achieving positive behavior change. However, without personal feedback from health professionals, self-monitoring is not usually enough to foster healthy behavioral change [15]. Our participants’ adherence to the biweekly self-weighing was higher among the telehealth group, 100%, in comparison to 96% for the in-person group, but this difference was not statistically significant. Likewise, the findings of the previous studies showed a higher adherence rate to self-weighing among the telehealth group [10,11]. Similarly, adherence to wearing the step counter was higher among the telehealth group, 75%, in comparison to the in-person group, 65%, but this did not reach a statistically significant level. Alencar et al.‘s study showed a similar higher adherence rate to wearing a step counter among the telehealth group [12].

Furthermore, subjects in the in-person group had a higher dropout rate of 21% compared to the telehealth group of 13% due to the higher convenience nature of the telehealth intervention, but this difference did not reach a statistically significant level. Likely, a randomized control trial revealed a higher dropout rate among the in-person group compared to the telehealth group [11]. Conversely, a male-only weight loss telehealth randomized control trial revealed a higher dropout rate among the telehealth group compared to the in-person group. This can be interpreted by the obviously higher number of counseling sessions for the telehealth group compared to the in-person group (15 versus 3 sessions, respectively) [8].

The two groups’ participants’ satisfaction levels were similar regarding “overall satisfaction with the allocated obesity management program” and “feel the assigned dietitian was helpful to them”, except for the question “feel the allocated obesity management program was helpful to lose weight?” The telehealth group’s satisfaction was higher, at 87%, compared to the in-person group’s 76%. The Barnason et al. study reported a high satisfaction rate for the weight management intervention among the telehealth group without comparing it with the in-person group [9]. On the contrary, Kee et al. [36] found lower patient satisfaction in the telehealth group compared to the in-person group. The main causes of this dissatisfaction were the age of more than 65 years, the absence of a common language between the health care provider and the patient, and management by different physicians. These factors were not available in the current study, i.e., all participants were <65 years old, both participants and healthcare providers speak Arabic, and the same healthcare provider managed all participants. Moreover, the availability of a stable network and the conservation of time and money were additional factors in the current study.

The concept of using telehealth in managing obesity and other chronic diseases is fast growing in Saudi Arabia based on well-developed communication infrastructure with the availability of 5G networking even in suburban and rural Saudi areas. Despite similar logistic infrastructure in Gulf countries, telehealth is still in its infancy [37]. Studies such as the current study may increase public and policymakers’ awareness of the value of telemedicine and telehealth services in the Gulf region [38].

This study is subject to limitations. First, it did not utilize a randomized controlled intervention trial design. Randomization techniques avoid any potential sources of bias and provide more robust evidence. However, randomization was impossible in this study because all healthcare providers knew about the study, and the availability of specific devices for the telehealth group made patients also aware of the intervention type. To reduce the bias of this limitation, we assessed the participants at the CAMS clinic (not at the KAIMRC, where the review board approved the study). This blinded the assessment to the participant’s treatment arm [39]. The second limitation is the 12-week short duration of the study. Future studies should extend the follow-up duration to evaluate the long-term efficacy and sustainability of the intervention. Third, we used a low-cost body composition analysis scale, and the validity has not yet been determined. We recommend replicating the study in the future using a valid body composition analysis scale to strengthen the reliability of the study findings. Furthermore, with a limited sample size, this study conducted many exploratory statistical tests, and the results must be interpreted cautiously.

## 5. Conclusions

Applying a telehealth obesity management program can bridge the gap and influence healthcare policies and practices in Saudi Arabia and beyond to reach and support patients struggling with obesity who may have limited access to traditional healthcare services. At the same time, ensuring that care via telehealth replicates the quality of in-person care can alter the healthcare delivery system and transform healthcare policies. Future studies investigating telehealth obesity programs on a long-term basis, how telehealth could be integrated into the healthcare systems, and its applicability at various socioeconomic levels are encouraged.

## Figures and Tables

**Table 1:** Baseline general characteristics of the in-person and telehealth groups.

Parameters		In Person (*n* = 29)	Telehealth (*n* = 33)	*p*-Value
Gender	Male	8 (27.6)	9 (27.3)	0.978
	Female	21 (72.4)	24 (72.7)	
Region	Riyadh	29 (100.0)	31 (93.9)	0.178
	Outside Riyadh	0 (0.0)	2 (6.1)	
Income	<10,000	3 (10.3)	10 (30.3)	0.117
	10,000–15,000	13 (44.8)	14 (42.4)	
	>15,000	13 (44.8)	9 (27.3)	
Smoking	Yes	3 (10.3)	3 (9.1)	0.868
	No	26 (89.7)	30 (90.9)	
Marital status	Single	18 (62.1)	20 (60.6)	0.507
	Married	8 (27.6)	11 (33.3)	
	Divorced	3 (10.3)	1 (3.0)	
	Widowed	0 (0.0)	1 (3.0)	
Academic level	Elementary	0 (0.0)	0 (0.0)	0.051
	Intermediate	1 (3.4)	0 (0.0)	
	High school	5 (17.2)	9 (27.3)	
	College	18 (62.1)	24 (72.7)	
	Higher education	5 (17.2)	0 (0.0)	
Readiness for change	Precontemplation	0 (0.0)	0 (0.0)	0.640
	Contemplation	0 (0.0)	1 (3.0)	
	Preparation	20 (69.0)	22 (66.7)	
	Action	9 (31.0)	10 (30.3)	
	Maintenance	0 (0.0)	0 (0.0)	
Dyslipidemia	No	27 (93.1)	32 (97.0)	0.749
	Yes	2 (6.9)	1 (3.0)	
Obesity	Mild (30–34.9)	15 (51.7)	18 (54.5)	0.791
	Moderate (35–39.9)	10 (34.5)	9 (27.3)	
	Severe (≥40)	4 (13.8)	6 (18.2)	
Osteoporosis	No	29 (100.0)	32 (97.0)	0.345
	Yes	0 (0.0)	1 (3.0)	
Asthma	No	27 (93.1)	30 (90.9)	0.752
	Yes	2 (6.9)	3 (9.1)	
One disease only	No	25 (86.2)	29 (87.9)	0.845
	Yes	4 (13.8)	4 (12.1)	
Poly morbidity	No	27 (93.1)	32 (97.0)	0.479
	Yes	2 (6.9)	1 (3.0)	

The *p*-value obtained from the Chi-square test and Fisher’s Exact test, *p* < 0.05, is considered significant.

**Table 2:** Age, anthropometric, and biochemical measurements of the telehealth and in-person groups at baseline.

	In-Person (*n* = 29)	Telehealth (*n* = 33)	*p*-Value
Age (years)	31.7 ± 11.8	32.3 ± 11.6	0.847
Weight (kg)	95.8 ± 13.9	95.7 ± 21.4	0.983
BMI (kg/m^2^)	35.1 ± 4.2	35.6 ± 5.5	0.703
Waist circumference (cm)	108.8 ± 11.3	107.0 ± 15.6	0.618
% body fat	42.6 ± 5.7	43.0 ± 4.8	0.802
Fasting glucose (mg/dL)	86.4 ± 19.7	90.7 ± 20.3	0.420
LDL (mg/dL)	127.5 ± 52.8	142.4 ± 54.4	0.297
Total cholesterol (mg/dL)	192.7 ± 71.5	206.3 ± 74.3	0.476
HDL cholesterol (mg/dL)	45.3 ± 18.0	44.2 ± 15.6	0.820
Triglycerides (mg/dL)	74.0 (45.0–111.0)	75.1 (40.0–118.5)	0.718
WBC (×10^3^/mL)	6.1 (5.4–7.6)	7.0 (5.7–9.1)	0.090
Hgb (g/dL)	11.3 (11.0–12.7)	11.0 (11.0–12.4)	0.862
MCV (fL)	82.0 (78.0–86.0)	77.0 (73.0–81.0)	0.003
MCH (Pg)	24.0 (22.6–28.0)	22.6 (21.0–24.0)	0.011
RDW (%)	16.0 (13.7–16.0)	16.0 (15.0–17.0)	0.016
RBC (×10^6^/mL)	4.8 (4.5–5.0)	5.0 (4.6–5.4)	0.099
Hct (%)	39.0 (36.5–40.6)	38.0 (36.2–43.2)	0.674
BUN (mg/dL)	8.5 (4.1–11.2)	8.6 (5.5–10.7)	0.767
Creatinine (mg/dL)	0.6 (0.5–0.6)	0.6 (0.6–0.7)	0.073
AST (U/L)	20.0 (14.0–24.0)	15.0 (13.5–16.5)	0.192
ALT(U/L)	19.0 (14.0–21.0)	13.0 (11.5–15.5)	0.210
Potassium (mmol/L)	4.6 (4.4–4.6)	4.3 (4.1–4.5)	0.020

Data presented as mean ± SD and Median (Quartile 1–Quartile 3) for normal and non-normal variables, respectively; *p*-value obtained from independent sample t-test and Mann–Whitney U test for normal and non-normal variables, respectively; *p* < 0.05 considered significant.

**Table 3:** Anthropometrics and blood pressure measurements, glucose, lipid, renal, hepatic, and vitamin D profiles within and between the two groups.

Parameters	In Person (*n* = 29)			Telehealth (*n* = 33)			Group	Interaction
	Baseline	12 Weeks	*p*-Value	Baseline	12 Weeks	*p*-Value	*p*-Value	*p*-Value
Anthropometrics								
Wt. (kg)	95.8 ± 13.9	90.7 ± 14.0	<0.001	95.7 ± 21.4	91.2 ± 21.2	<0.001	0.965	0.486
Wt. loss	5.2 ± 3.7			4.6 ± 3.1			0.486	
% wt. loss	5.4 ± 4.2			4.8 ± 3.0			0.532	
Wt. loss (5%)	44.80%			48.50%			0.773	
BMI (kg/m^2^)	35.1 ± 4.2	33.2 ± 4.1	<0.001	35.6 ± 5.4	33.9 ± 5.4	<0.001	0.650	0.210
WC (cm)	108.8 ± 11.3	102.4 ± 11.3	<0.001	107.0 ± 15.6	101.8 ± 15.0	<0.001	0.763	0.622
% body fat	42.6 ± 5.7	41.0 ± 6.0	<0.001	43.0 ± 4.8	41.7 ± 4.9	<0.001	0.745	0.131
Lipids and Glucose								
Fasting glucose (mg/dL)	86.4 ± 19.7	88.6 ± 14.4	0.644	90.7 ± 20.3	94.7 ± 14.8	0.067	0.348	0.405
LDL (mg/dL)	127.5 ± 52.8	129.6 ± 48.4	0.608	142.4 ± 54.4	142.8 ± 44.1	0.567	0.258	0.989
Total cholesterol (mg/dL)	192.7 ± 71.5	191.8 ± 59.3	0.796	206.3 ± 74.3	207.5 ± 54.6	0.681	0.373	0.928
HDL (mg/dL)	45.3 ± 18.0	44.3 ± 13.8	0.738	44.2 ± 15.6	46.0 ± 12.8	0.297	0.958	0.639
Triglycerides (mg/dL)	74.0 (45.0–111.0)	72.0 (55.0–112.0)	0.332	75.1 (40.0–118.5)	79.0 (65.0–130.0)	0.152	0.489	0.812
Renal, Liver Profile, and Vitamin D								
GFR (mL/min/1.73 m^2^)	124.4 ± 26.8	113	0.672	114.6 ± 9.7	121.0 ± 4.0	0.258	0.121	0.539
Creatinine (mg/dL)	0.6 ± 0.1	0.6 ± 0.1	0.204	0.6 ± 0.1	0.7 ± 0.2	0.271	0.083	0.878
BUN (mg/dL)	8.5 (4.1–11.2)	9.4 (6.8–11.4)	0.186	8.6 (5.5–10.7)	10.4 (7.6–12.3)	0.002	0.601	0.205
Potassium (mmol/L)	4.5 ± 0.2	4.3 ± 0.5	1.000	4.3 ± 0.2	4.3 ± 0.1	0.853	0.454	0.879
Uric acid (mg/dL)	5.7 ± 2.0	5.8 ± 2.1	0.390	5.9 ± 2.5	6.2 ± 1.9	0.545	0.310	0.345
Aspartate aminotransferase (u/L)	22.1 ± 10.3	21.7 ± 7.0	0.477	16.1 ± 6.1	16.3 ± 2.6	0.801	0.439	0.681
Alanine transaminase (u/L)	19.0 (14.0–21.0)	19.0 (11.0–19.0)	0.773	13.0 (11.5–15.5)	15.5 (13.0–21.5)	0.652	0.986	0.947
Vitamin D 25 OH (ng/mL)	32.9 (22.9–54.5)	37.0 (27.2–39.5)	0.525	37.0 (21.6–61.3)	40.0 (31.0–88.0)	0.253	0.933	0.280
Blood pressure								
Systolic	132.8 ± 20.7	127.3 ± 12.7	0.276	127.3 ± 15.3	124.2 ± 28.5	0.53	0.349	0.723
Diastolic	73.5 ± 12.7	72.0 ± 10.2	0.670	70.0 ± 8.7	72.2 ± 17.3	0.517	0.536	0.450

Note: Data presented as Mean ± SD for continuous normal variables and N (%) for categorical variables; from repeated measures, ANOVA, *p* < 0.05 is considered significant.

**Table 4:** Physical activity levels within and between the two groups.

Physical Activity (PA)		In-Person (*n* = 29)			Telehealth (*n* = 33)			Groups	Interactions
		Baseline	12 Weeks	*p*-Value	Baseline	12 Weeks	*p*-Value	*p*-Values	*p*-Values
Light PA 30 min (e.g., walking and housework)	At least once a week	19 (65.5)	4 (13.8)	<0.001	20 (60.6)	7 (21.2)	<0.001	0.33	0.302
	More than once a week	10 (34.5)	25 (86.2)		13 (39.4)	26 (78.8)			
Moderate PA (e.g., brisk walking, swimming, cycling, or farm work)	At least once a week	24 (82.8)	19 (65.5)	0.18	30 (90.9)	23 (69.7)	0.039	0.776	0.515
	More than once a week	5 (17.2)	10 (34.5)		3 (9.1)	10 (30.3)			
Vigorous PA (e.g., running, playing ball, or strenuous work)	At least once a week	26 (89.7)	24 (82.7)	0.625	29 (87.9)	25 (75.7)	0.289	0.583	0.764
	More than once a week	3 (10.3)	5 (17.2)		4 (12.1)	8 (24.2)			

Note: Data are presented as N (%); *p*-value obtained from generalized estimating equations (GEE); *p* < 0.05 considered significant.

**Table 5:** Sleeping habits and macronutrient intake within and between the two groups.

Lifestyle Parameters	In Person (*n* = 29)			Telehealth (*n* = 33)			Group	Interaction
	Baseline	12 Weeks	*p*-Value	Baseline	12 Weeks	*p*-Value	*p*-Value	*p*-Value
Sleeping Habits								
Total hours sleep	5.7 ± 2.3	4.1 ± 1.6	0.002	5.6 ± 2.5	4.4 ± 1.3	0.010	0.793	0.574
Hours of sleep at night	4.5 ± 2.1	3.5 ± 1.1	0.028	4.8 ± 2.3	3.8 ± 1.0	0.013	0.318	0.913
Time to fall asleep (minutes)	3.8 ± 1.5	3.2 ± 1.4	0.04	4.1 ± 1.1	3.4 ± 1.3	0.010	0.454	0.774
Food Intake								
Fruit intake	1.4 ± 0.3	1.5 ± 0.3	0.012	1.5 ± 0.3	1.6 ± 0.2	0.005	0.159	0.855
Vegetable intake	1.9 ± 0.5	2.1 ± 0.4	0.083	1.9 ± 0.4	2.2 ± 0.4	<0.001	0.526	0.104
Meat and meat substitute intake	1.7 ± 0.2	1.6 ± 0.2	0.09	1.7 ± 0.2	1.7 ± 0.2	0.409	0.105	0.068
Dairy products intake	1.3 ± 0.3	1.4 ± 0.3	0.452	1.4 ± 0.3	1.4 ± 0.2	0.495	0.475	0.938
Carbohydrate intake	1.5 ± 0.3	1.3 ± 0.2	0.022	1.5 ± 0.2	1.4 ± 0.2	0.036	0.388	0.640
Fat intake	1.6 ± 0.4	1.7 ± 0.3	0.567	1.7 ± 0.4	1.7 ± 0.3	0.287	0.468	0.951

Note: Data presented as Mean ± SD; *p*-value obtained from repeated measures ANOVA; *p* < 0.05 considered significant.

## Data Availability

The corresponding author may provide the data described in this research upon request.

## Supplementary Materials

The following supporting information can be downloaded at https://www.mdpi.com/article/10.3390/healthcare12212190/s1, Table S1: Intervention during study visits in both groups; Table S2: Dietary intake of macronutrients using three-day food record within and between the two groups; Table S3: Dietary intake of vitamins using three-day food record within and between the two groups; Table S4: Dietary intake of minerals and trace elements using three-day food record within and between the two groups; Table S5: Eating habits characteristics within and between the two groups.
