# Supplementary material for: The Efficacy of Telehealth Versus In-Person Management Delivery in Adult Patients with Obesity
Source: Healthcare (Basel). 2024 Nov 4;12(21):2190. doi: 10.3390/healthcare12212190 (PMC11545763; doi:10.3390/healthcare12212190)
Supplement: Supplementary file 1 [file healthcare-12-02190-s001.zip › healthcare-3281415-supplementary.pdf]

Table S1. Intervention during study visits in both groups.

	In-person intervention	Telehealth intervention
1st physical visit (60min)	• Socio-demographic data questionnaire<br>• Vital signs: blood pressure measurement.<br>• Anthropometrics: Wt., Ht., BMI, WC, % fat body.<br>• Biochemical tests: Fasting BS, TC., LDL, HDL-C, creatinine, BUN, GFR, CBC, ALS, ALT, K, uric acid, vitamin D.<br>• Medical history & medication intake<br>• Dietary: 3 days food record, food frequency questionnaire, nutritional assessment.<br>• Lifestyle data: physical activity level, sleeping pattern, sun exposure questionnaire, & 90 days daily step count record.<br>• Assess patient readiness for change.<br>• Patient was given wireless body weighing & body composition scale & wearable tracker for physical activity.<br>• Education about eating a well-balanced Mediterranean-style diet: about half the plate should contain vegetables, about one-quarter should include protein-rich (meat, fish, cheese, and legumes), and the last quarter should include starchy food, including cereals and grains.	• Socio-demographic data questionnaire<br>• Vital signs: blood pressuremeasurement.<br>• Anthropometrics: Wt., Ht., BMI, WC, % fat body.<br>• Biochemical tests: Fasting BS, TC., LDL, HDL-C, creatinine, BUN, GFR, CBC, ALS, ALT, K, uric acid, vitaminD.<br>• Medical history & medication intake<br>• Dietary: 3 days food record, food frequency questionnaire, nutritionalassessment.<br>• Lifestyle data: physical activity level,sleeping pattern, sun exposure questionnaire, & 90 days daily step count record.<br>• Assess patient readiness for change.<br>• Patient was given wireless body weighing & body composition scale &wearable tracker for physical activity.<br>• Education about eating a well-balanced Mediterranean-style diet: about half the plate should contain vegetables, about one-quarter should include protein-rich (meat, fish, cheese, and legumes), and the last quarter should include starchy food, including cereals and grains.
Follow-up visits No.2 (30 min.)	• Patients received reminder text messages to measure their wt. and %body fat and prepare two two-week records of daily step count before the appointment.<br>• Nutritional counseling session with the dietitian at the clinic.<br>• Education about personalized meal plan based on Mifflin St Joer equation with targeted energy deficit of 600 Calories per day.<br>• Encourage aerobic physical activity for 150 to 300 minutes per week and to increase daily living activities and the number of steps to 5000-10000 steps per day.	• Patients received reminder text messages to measure their weight. % bodyfat, and prepare a two-week record of daily step count before the appointment.<br>• Nutritional counseling session with dietitian via videoconferencing.<br>• Education about personalized meal plans based on the Mifflin St Joer equation with a targeted energy deficit of 600 Calories per day.<br>• Encourage aerobic physical activity for 150 to 300 minutes per week, and to increase daily living activities and the number of steps to 5000-10000 steps per day.

Follow-up visit no.3 (30 min.)	• Patients received reminder text messages to measure their weight and body fat, and prepare two weeks record of daily steps count before the appointment.<br>• Nutritional counseling session with the dietitian at the clinic.<br>• Education about how to achieve satiety through drinking water before meals, drinking water when they feel hungry (total of 2 litters of water per day), start meals with vegetables, then protein, encourage moderate amount of healthy fat, encourage complex carbohydrate intake, instead of simple carbohydrate, encourage mindful eating eat slowly and chew food thoroughly in a quiet environment.	• Patients received reminder text messages to measure their weight and bodyfat and prepare 2 2-week records of daily step count before the appointment.<br>• Nutritional counseling session with dietitian via videoconferencing.<br>• Education about how to achieve satiety through drinking water before meals, drinking water when they feel hungry (total of 2 litters of water per day), starting meals with vegetables, then protein, encouraging the moderate amount of healthy fat, encouraging complex carbohydrate intake, instead of simple carbohydrate, encourage mindful eating eat slowly and chew food thoroughly in quiet environment.
Follow-up visits no.4 (30 min.)	• Patients received reminder text message to measure their wt., %body fat, and prepare 2 weeks record of daily steps count before the appointment.<br>• Nutritional counselling session with the dietitian at the clinic.<br>• Education about healthy snacks e.g., replace chips with nuts & popcorn, and healthy food choices at restaurants e.g., replace white sauce pasta with red sauce pasta, replace peperoni pizza with vegetables & meat pizza.	• Patients received reminder text message to measure their wt., %bodyfat, and prepare 2 weeks record of daily steps count before the appointment.<br>• Nutritional counselling session with dietitian via videoconferencing.<br>• Education about healthy snacks e.g., replace chips with nuts & popcorn, and healthy food choices at restaurants e.g., replace white sauce pasta with red sauce pasta, replace peperoni pizza with vegetables & meat pizza.
Follow-up visits no.5 (30 min.)	• Patients received reminder text message to measure their wt., %body fat, and prepare 2 weeks record of daily steps count before the appointment.<br>• Nutritional counselling session with the dietitian at the clinic.<br>• Education about what to do after eating unhealthy meal: take a walk for 30 min., avoid lying down, avoid rice, bread, pasta during the next day, eat a lot of fruit, vegetables & proteins to	• Patients received reminder text message to measure their wt., %bodyfat, and prepare 2 weeks record of daily steps count before the appointment.<br>• Nutritional counselling session with dietitian via videoconferencing.<br>• Education about what to do after eating unhealthy meal: take a walk for 30 min., avoid lying down, avoid rice, bread, pasta during the next day, eat a lot of fruit, vegetables & proteins to reset the taste sensation

	reset the taste sensation & regulate hunger & satiety hormones, sleep for 7 hours, don't measure your weight.	& regulate hunger & satiety hormones, sleep for 7 hours, don't measure your weight.
Follow-up visits no.6 (30 min.)	• Patients received reminder text message to measure their wt., %body fat, and prepare 2 weeks record of daily steps count before the appointment.<br>• Nutritional counselling session with the dietitian at the clinic.<br>• Education about weight maintenance tips: weekly weight measurement, avoid processed food & sweets, avoid drinking calories, early meal prep for work, maintain physical activity routine, avoid night eating.	• Patients received reminder text message to measure their wt., %body fat, and prepare 2 weeks record of daily steps count before the appointment.<br>• Nutritional counselling session with dietitian via videoconferencing.<br>• Education about weight maintenance tips: weekly weight measurement, avoid processed food & sweets, avoid drinking calories, early meal prep for work, maintain physical activity routine, avoid night eating.
Final visit physical: number 7 (60 min.)	• Vital signs: blood pressure measurement.<br>• Anthropometrics: wt., ht., BMI, WC, % body fat.<br>• Biochemical tests: Fasting BS, TC.,LDL, HDL, creatinine, BUN, CBC.<br>• Medical history & medication intake<br>• Dietary: 3 days food record, food frequency questionnaire.<br>• Lifestyle data: physical activity level, sleeping pattern, sun exposure questionnaire.<br>• Patient satisfaction survey & 90 days daily step count record.<br>• Patients returned the wireless body weighing & body composition scale & the wearable tracker for physical activity.	• Vital signs: blood pressure measurement.<br>• Anthropometrics: wt., ht., BMI, WC, % body fat.<br>• Biochemical tests: Fasting BS, TC.,LDL, HDL, creatinine, BUN, CBC.<br>• Medical history & medication intake<br>• Dietary: 3 days food record, food frequency questionnaire.<br>• Lifestyle data: physical activity level, sleeping pattern, sun exposure questionnaire.<br>• Patient satisfaction survey & 90 days daily step count record.<br>• Patients returned the wireless body weighing & body composition scale & the wearable tracker for physical activity

Table S2. Dietary intake of macronutrients using a 3-day food record within and between the two groups.

Dietary Intake	In-person (n=29)			Telehealth (n=33)			Group	Interaction
	Baseline	12 weeks	P-value	Baseline	12 weeks	P-value	P-value	P-value
Calories (kcal)	1074.6 (894.8 - 1762.1)	984.8 (766.2 - 1268.3)	0.268	1110.1 (911.0 - 1520.0)	819.1 (704.0 - 1285.2)	0.049	0.746	0.568
Fat Cals (kcal)	454.2 (293.7 - 657.9)	298.3 (222.4 - 407.3)	0.043	438.8 (323.3 - 564.9)	278.0 (211.9 - 374.1)	0.021	0.892	0.892
Saturated fat Cals(kcal)	147.1 (73.7 - 218.8)	70.1 (52.7 - 114.2)	0.026	139.5 (103.7 - 192.8)	89.3 (59.8 - 115.4)	0.036	0.726	0.864
Protein (g)	56.5 (44.6 - 87.5)	52.0 (42.8 - 62.6)	0.601	58.5 (31.8 - 85.3)	44.3 (38.0 - 77.2)	0.745	0.649	0.877
Carbohydrate (g)	121.9 (97.0 - 173.0)	106.1 (85.2 - 144.3)	0.595	122.0 (99.8 - 177.0)	99.9 (73.0 - 134.0)	0.102	0.873	0.451
Total Fibers (g)	7.6 (5.2 - 14.5)	11.6 (6.1 - 13.1)	0.425	10.1 (5.3 - 13.5)	8.8 (5.1 - 11.4)	0.949	0.942	0.593
Total Soluble Fibers (g)	0.1 (0.0 - 0.2)	0.1 (0.0 - 0.5)	0.14	0.1 (0.0 - 0.3)	0.0 (0.0 - 0.4)	0.451	0.975	0.114
Sugar (g)	46.3 (26.0 - 63.0)	33.8 (14.5 - 50.5)	0.222	50.3 (30.9 - 70.1)	30.6 (16.3 - 40.7)	0.025	0.595	0.493
Added Sugar (g)	9.7 (4.0 - 23.2)	7.3 (2.7 - 15.0)	0.241	11.9 (4.1 - 30.0)	6.0 (3.3 - 10.3)	0.012	0.891	0.353
Mono Saccharide (g)	2.4 (0.0 - 6.4)	2.1 (0.1 - 8.8)	0.869	1.4 (0.0 - 7.6)	1.1 (0.0 - 6.4)	0.7	0.529	0.703
Disaccharide (g)	0.6 (0.1 - 2.8)	0.8 (0.0 - 3.3)	0.636	0.7 (0.1 - 3.3)	0.3 (0.0 - 2.1)	0.3	0.746	0.295
Oligosaccharide (g)	64.3 (47.8 - 96.9)	63.2 (51.6 - 83.5)	0.927	76.1 (51.1 - 87.5)	53.1 (37.9 - 68.1)	0.168	0.456	0.307
Fat (g)	50.5 (32.9 - 73.1)	33.1 (24.7 - 45.3)	0.043	48.8 (35.9 - 62.8)	30.9 (23.5 - 41.6)	0.022	0.897	0.902
Saturated Fat (g)	16.3 (8.2 - 24.3)	7.8 (5.9 - 12.7)	0.028	15.5 (11.5 - 21.4)	9.9 (6.6 - 12.8)	0.041	0.736	0.851

Monounsaturated Fat (g)	12.2 (6.6 - 17.2)	8.8 (5.1 - 10.6)	0.089	11.4 (8.6 - 17.8)	8.5 (5.2 - 11.2)	0.087	0.578	0.957
Polyunsaturated Fat (g)	6.1 (3.3 - 8.8)	5.0 (3.8 - 7.2)	0.322	5.2 (3.7 - 8.2)	4.3 (3.2 - 5.3)	0.307	0.83	0.989
Trans Fat (g)	0.1 (0.0 - 0.2)	0.0 (0.0 - 0.1)	0.697	0.1 (0.0 - 0.2)	0.0 (0.0 - 0.2)	0.326	0.805	0.338
Cholesterol (mg)	238.1 (174.3 - 312.7)	194.2 (155.8 - 299.0)	0.705	242.4 (146.5 - 344.0)	219.8 (120.9 - 358.0)	0.664	0.96	0.98
Water content of food (ml)	467.4 (386.1 - 589.4)	451.7 (332.1 - 607.4)	0.874	489.7 (374.9 - 665.6)	464.1 (292.2 - 642.4)	0.563	0.949	0.607

Note: Data presented as Median (Quartile 1 – Quartile 3); Log Transformation was used to achieve approximate normality; P-value obtained from repeated measures ANOVA; P<0.05 considered significant.

Table S3. Dietary Intake of vitamins using 3days food record within and between the two groups.

Dietary Intake	In-Person (n=29)			Telehealth (n=33)			Group	Interaction
	**Baseline**	**12 weeks**	**P-value**	**Baseline**	**12 weeks**	**P-value**	**P-value**	**P-value**
Vitamin A Rare (mcg)	152.0 (84.1 - 250.9)	178.0 (75.5 - 302.2)	0.455	255.8 (148.5 - 403.1)	228.5 (144.5 - 322.3)	0.618	**0.029**	**0.377**
Carotene (mcg)	23.8 (7.0 - 73.3)	26.3 (4.3 - 161.7)	0.965	35.3 (5.4 - 79.6)	33.1 (5.7 - 68.5)	0.864	0.889	0.881
Retinol (mcg)	107.3 (44.1 - 189.3)	83.5 (46.5 - 143.4)	0.913	167.5 (60.7 - 284.0)	96.1 (44.6 - 163.7)	0.105	0.131	0.229
Beta Carotene	242.5 (62.7 -	383.6 (141.4	0.670	536.3 (61.7 -	1327 (204.2 -	0.154	0.157	**0.495**

(mcg)	- 672.5)	1331.8)		1330.3)	2282.6)			
Vitamin B1 (mg)	0.5 (0.3 - 0.7)	0.5 (0.3 - 0.6)	0.928	0.5 (0.4 - 0.7)	0.4 (0.3 - 0.7)	0.851	0.634	0.949
Vitamin B2 (mg)	0.7 (0.4 - 1.1)	0.6 (0.5 - 0.8)	0.637	0.9 (0.7 - 1.2)	0.6 (0.4 - 0.9)	0.594	0.219	0.979
Vitamin B3 (mg)	9.1 (6.2 - 17.0)	11.4 (7.2 - 15.8)	0.461	10.5 (6.7 - 17.5)	8.4 (6.0 - 13.5)	0.071	0.811	0.076
Vitamin B3 NE (mg)	11.5 (7.0 - 17.4)	12.5 (9.6 - 17.7)	0.490	12.9 (9.3 - 18.8)	10.7 (6.7 - 15.7)	0.220	0.777	**0.180**
Vitamin B6 (mg)	0.7 (0.4 - 1.0)	0.6 (0.5 - 1.1)	0.899	0.8 (0.6 - 1.1)	0.7 (0.5 - 1.1)	0.750	0.904	0.898
Vitamin B12 (mcg)	1.5 (0.8 - 3.0)	1.3 (0.7 - 2.2)	0.900	2.2 (1.0 - 3.5)	1.6 (1.1 - 2.5)	0.386	0.150	**0.612**
Biotin (mcg)	6.9 (2.3 - 11.0)	7.8 (4.6 - 10.3)	0.811	6.6 (2.7 - 9.1)	8.7 (2.9 - 14.5)	0.053	0.631	0.258
Vitamin C (mg)	10.8 (6.8 - 42.0)	14.5 (7.0 - 45.7)	0.965	21.0 (7.7 - 52.4)	10.2 (5.0 - 40.9)	0.102	0.497	0.270
Vitamin D (mcg)	1.6 (0.3 - 4.0)	2.1 (0.8 - 3.6)	0.924	2.8 (1.1 - 4.5)	2.0 (0.8 - 3.1)	0.554	0.247	0.735
Vitamin E-alpha Tocopherol (mg)	2.2 (1.2 - 3.9)	2.1 (1.5 - 3.3)	0.640	2.4 (1.7 - 3.4)	1.9 (1.5 - 2.7)	0.173	0.557	0.544
Folate (mcg)	125.4 (73.2 - 184.9)	149.3 (97.3 - 201.4)	0.301	131.8 (103.5 - 184.1)	98.5 (76.5 - 157.3)	0.649	0.975	0.870
Folate DFE (mcg DFE)	161.8 (97.0	165.4 (127.0 -	0.253	178.6 (138.9	121.9 (95.4 -	0.472	0.876	0.186

	- 236.3)	240.1)		- 223.7)	185.0)			
Vitamin K (mcg)	23.6 (11.4 - 29.8)	25.1 (7.3 - 43.4)	0.739	16.8 (9.5 - 43.3)	15.4 (8.7 - 38.1)	0.940	0.776	0.770
Pantothe nic acid (mg)	1.3 (0.8 - 2.3)	1.2 (0.8 - 2.0)	0.968	1.3 (0.7 - 1.7)	1.5 (0.8 - 2.2)	0.220	0.898	**0.385**

Note: Data presented as Median (Quartile 1 – Quartile 3); Log Transformation was used to achieve approximate normality; P-value obtained from repeated measures ANOVA; P<0.05 considered significant.

Table S4. Dietary Intake of minerals and trace elements using 3 days food record within and between the two groups.

**Dietary Intake**	**In-Person (n=29)**			**Telehealth (n=33)**			**P-value**	**P-value**
	**Baseline**	**Final**	**P-value**	**Baseline**	**Final**	**P-value**		
Calcium (mg)	464.6 (178.8 - 708.5)	298.0 (154.9 - 412.4)	0.178	377.1 (222.7 - 585.4)	285.7 (219.1 - 414.9)	0.615	0.598	0.526
Chromium (mcg)	0.9 (0.6 - 2.1)	0.7 (0.3 - 1.7)	0.984	0.9 (0.4 - 1.3)	0.7 (0.4 - 1.7)	0.283	0.427	0.410
Copper (mg)	0.5 (0.3 - 0.6)	0.4 (0.3 - 0.6)	0.792	0.5 (0.3 - 0.9)	0.4 (0.3 - 0.7)	0.278	0.184	0.384
Fluoride (mg)	0.1 (0.0 - 0.2)	0.1 (0.0 - 0.2)	0.646	0.1 (0.0 - 0.2)	0.0 (0.0 - 0.1)	**0.025**	0.574	0.060
Iodine (mcg)	13.9 (0.9 - 29.4)	15.3 (0.8 - 26.2)	0.983	17.3 (1.4 - 29.5)	22.3 (1.2 - 44.2)	0.244	0.311	0.432
Iron (mg)	6.3 (4.6 - 7.4)	5.4 (3.8 - 6.4)	0.841	5.9 (4.8 - 8.0)	4.5 (3.8 - 6.5)	0.222	0.874	0.483
Magnesium (mg)	97.1 (89.0 - 138.3)	100.7 (75.0 - 124.3)	0.656	114.9 (76.4 - 152.3)	103.9 (70.7 - 142.1)	0.556	0.969	0.933
Manganese (mg)	0.3 (0.2 - 0.7)	0.4 (0.2 - 0.5)	0.672	0.2 (0.1 - 0.4)	0.2 (0.1 - 0.6)	0.806	0.460	0.634

Molybdenum (mcg)	6.7 (4.4 - 12.6)	9.4 (5.2 - 12.9)	0.207	6.6 (3.1 - 11.9)	13.3 (8.0 - 19.4)	0.052	0.149	0.655
Phosphorus (mg)	501.9 (336.0 - 717.6)	443.6 (352.0 - 666.2)	0.679	574.1 (440.8 - 912.6)	479.8 (355.0 - 697.7)	0.465	0.663	0.837
Potassium (mg)	1180.3 (941.3 - 1440.0)	1083.2 (853.5 - 1559.2)	0.582	1474.8 (913.2 - 1920.7)	1168.5 (717.8 - 1412.9)	0.245	0.559	**0.684**
Selenium (mcg)	37.2 (31.8 - 74.2)	53.9 (38.3 - 73.4)	0.418	50.0 (32.2 - 86.3)	42.8 (29.2 - 66.8)	0.701	0.970	0.395
Sodium (mg)	1742.4 (1341.5 - 2349.1)	1603.2 (1117.2 - 1880.8)	0.428	1860.4 (1234.6 - 2503.1)	1299.4 (900.5 - 1723.2)	0.357	0.450	0.951
Zinc (mg)	3.8 (2.7 - 6.3)	3.0 (2.4 - 4.4)	0.461	4.2 (2.9 - 6.8)	3.6 (2.4 - 4.9)	0.565	0.355	0.891
Omega 3 (g)	0.5 (0.2 - 0.8)	0.4 (0.3 - 0.7)	0.531	0.4 (0.3 - 0.8)	0.3 (0.2 - 0.5)	0.830	0.976	0.759
Omega 6 (g)	5.4 (2.5 - 7.2)	4.2 (2.7 - 6.0)	0.195	4.6 (3.0 - 6.7)	3.1 (2.5 - 4.7)	0.160	0.784	0.975
Acetyl-L-carnitine (g)	0.0 (0.0 - 0.0)	0.0 (0.0 - 0.0)	0.876	0.0 (0.0 - 0.0)	0.0 (0.0 - 0.0)	0.175	0.409	0.294
Caffein (mg)	40.0 (4.2 - 80.4)	40.0 (0.0 - 82.0)	0.514	42.0 (21.0 - 78.9)	5.8 (0.0 - 54.0)	**<0.001**	0.791	0.015
Chlorophyllin (mg)	159.6 (80.8 - 236.4)	172.1 (87.1 - 243.1)	0.644	195.0 (113.1 - 243.3)	162.9 (103.7 - 295.1)	0.805	0.381	0.614

Note: Data presented as Median (Quartile 1 – Quartile 3); Log Transformation was used to achieve approximate normality; P-value obtained from repeated measures ANOVA; P<0.05 considered significant.

Table S5. Eating habits characteristics within and between the two groups.

Questions	Physical			Virtual			P-value
	Baseline	Follow-up	P	Baseline	Follow-up	P	
How many meals do you eat during the day don’t count Snacks							
mean ± SD	2.0 ± 0.6	2.1 ± 0.5	0.232	2.0 ± 0.7	2.2 ± 0.6	0.354	0.736
In which of the main meals do you usually eat the largest amount of carbohydrates and sugars							
Breakfast	3 (11.5)	5 (19.2)	0.607	2 (6.3)	7 (21.9)	0.027	0.104
Lunch	16 (61.5)	14 (53.8)		19 (59.4)	23 (71.9)		
Dinner	7 (26.9)	7 (26.9)		11 (34.4)	2 (6.3)		
In which of the main meals do you usually eat the most protein							
Breakfast	3 (11.1)	6 (22.2)	0.413	4 (12.5)	5 (15.6)	0.609	0.788
Lunch	17 (63.0)	18 (66.7)		18 (56.3)	27 (84.4)		
Dinner	7 (25.9)	3 (11.1)		10 (31.3)	0		
In which of the main meals do you usually eat the largest amount of fats							
Breakfast	2 (7.7)	3 (11.5)	0.140	4 (13.3)	4 (13.3)	0.027	0.622
Lunch	12 (46.2)	17 (65.4)		14 (46.7)	23 (76.7)		
Dinner	12 (46.2)	6 (23.1)		12 (40.0)	3 (10.0)		
Do you eat breakfast							
Always	4 (13.8)	1 (3.4)	0.265	5 (15.2)	0	0.609	0.988
Often	5 (17.2)	2 (6.9)		6 (18.2)	5 (15.2)		
Sometimes	2 (6.9)	7 (24.1)		6 (18.2)	9 (27.3)		
Rarely	5 (17.2)	2 (6.9)		9 (27.3)	2 (6.1)		
Never	13 (44.8)	17 (58.6)		7 (21.2)	17 (51.5)		
Do you sleep after eating a main meal (2hours or less apart)?							
Always	9 (31.0)	22 (75.9)	0.055	11 (33.3)	22 (66.7)	0.088	0.593
Often	7 (24.1)	3 (10.3)		3 (9.1)	4 (12.1)		
Sometimes	4 (13.8)	2 (6.9)		11 (33.3)	5 (15.2)		
Rarely	4 (13.8)	1 (3.4)		6 (18.2)	2 (6.1)		
Never	5 (17.2)	1 (3.4)		2 (6.1)	0		
If you sleep after eating a main meal? what is the meal?							

Breakfast	0	0	0.625	0	1 (10.0)	1.00	0.181
Lunch	3 (75.0)	2 (40.0)		1 (10.0)	1 (10.0)		
Dinner	1 (25.0)	3 (60.0)		9 (90.0)	8 (80.0)		
Does your desire to eat certain foods wake you up from your sleep or cause you insomnia or inability to sleep?							
Always	22 (75.9)	25 (86.2)	0.549	28 (87.5)	28 (87.5)	1.000	0.691
Often	3 (10.3)	2 (6.9)		2 (6.3)	2 (6.3)		
Sometimes	2 (6.9)	2 (6.9)		1 (3.1)	2 (6.3)		
Rarely	1 (3.4)	0		1 (3.1)	0		
Never	1 (3.4)	0		0	0		
Do you eat snacks							
Always	1 (3.4)	1 (3.4)	0.315	3 (9.1)	2 (6.1)	0.181	0.103
Often	4 (13.8)	7 (24.1)		7 (21.2)	4 (12.1)		
Sometimes	4 (13.8)	5 (17.2)		2 (6.1)	9 (27.3)		
Rarely	3 (10.3)	5 (17.2)		5 (15.2)	1 (3.0)		
Never	17 (58.6)	11 (37.9)		16 (48.5)	17 (51.5)		
How many snacks do you eat during the day							
mean ± SD	1.8 ± 0.8	1.6 ± 0.7	0.460	1.8 ± 0.9	1.8 ± 0.8	0.730	0.452
On average how many cups of water you drink?							
mean ± SD	3.1 ± 1.3	4.1 ± 1.2	0.001	3.0 ± 1.3	4.5 ± 1.2	0.001	0.588
I usually drink water when..?							
Always	6 (20.7)	0	0.48	7 (21.2)	1 (3.0)	0.512	0.301
Often	4 (13.8)	1 (3.4)		5 (15.2)	4 (12.1)		
Sometimes	2 (6.9)	2 (6.9)		2 (6.1)	0		
Rarely	9 (31.0)	16 (55.2)		7 (21.2)	5 (15.2)		
Never	8 (27.6)	10 (34.5)		12 (36.4)	23 (69.7)		
Drink Water: Just before eating							
Always	14 (48.3)	2 (6.9)	0.006	11 (33.3)	6 (18.2)	0.402	0.143
Often	2 (6.9)	1 (3.4)		2 (6.1)	3 (9.1)		
Sometimes	3 (10.3)	10 (34.5)		8 (24.2)	9 (27.3)		
Rarely	7 (24.1)	2 (6.9)		4 (12.1)	3 (9.1)		
Never	3 (10.3)	14 (48.3)		8 (24.2)	12 (36.4)		
Drink water: before sleep							
Always	10 (34.5)	6 (20.7)	0.242	3 (9.1)	4 (12.1)	0.195	0.298
Often	3 (10.3)	1 (3.4)		3 (9.1)	3 (9.1)		
Sometimes	4 (13.8)	4 (13.8)		7 (21.2)	6 (18.2)		

Rarely	4 (13.8)	2 (6.9)		9 (27.3)	3 (9.1)		
Never	8 (27.6)	16 (55.2)		11 (33.3)	17 (51.5)		
Drink water: after waking up							
Always	6 (20.7)	0		2 (6.1)	2 (6.1)	0.176	0.069
Often	3 (10.3)	0		2 (6.1)	1 (3.0)		
Sometimes	2 (6.9)	3 (10.3)	0.057	5 (15.2)	3 (9.1)		
Rarely	0	0		7 (21.2)	3 (9.1)		
Never	18 (62.1)	26 (89.7)		17 (51.5)	24 (72.7)		
Is salt added during the cooking process of different foods							
Never	0	1 (3.4)		1 (3.0)	2 (6.1)	1.00	0.297
Rarely	0	0		2 (6.1)	1 (3.0)		
Sometimes	4 (13.8)	1 (3.4)	1.00	0	0		
Often	1 (3.4)	2 (6.9)		1 (3.0)	2 (6.1)		
Always	24 (82.8)	25 (86.2)		29 (87.9)	28 (84.8)		
Is salt added during to food on the table before eating							
Never	23 (79.3)	26 (89.7)		28 (84.8)	27 (81.8)	1.00	0.572
Rarely	1 (3.4)	0		0	1 (3.0)		
Sometimes	1 (3.4)	0	1.00	0	0		
Often	1 (3.4)	0		1 (3.0)	1 (3.0)		
Always	3 (10.3)	3 (10.3)		4 (12.1)	4 (12.1)		
On average how many spoons of sugar	1.6 ± 0.7	1.2 ± 0.4	0.037	1.7 ± 1.0	1.7 ± 1.5	1.00	0.454
